# PatternJ: an ImageJ toolset for the automated and quantitative analysis of regular spatial patterns found in sarcomeres, axons, somites, and more

**DOI:** 10.1242/bio.060548

**Published:** 2024-06-18

**Authors:** Mélina Baheux Blin, Vincent Loreau, Frank Schnorrer, Pierre Mangeol

**Affiliations:** Aix Marseille Université, CNRS, Institut de Biologie du Développement de Marseille, IBDM-UMR7288, Marseille 13009, France

**Keywords:** Imagej, Automated analysis, Axon, Regular patterns, Sarcomeres

## Abstract

Regular spatial patterns are ubiquitous forms of organization in nature. In animals, regular patterns can be found from the cellular scale to the tissue scale, and from early stages of development to adulthood. To understand the formation of these patterns, how they assemble and mature, and how they are affected by perturbations, a precise quantitative description of the patterns is essential. However, accessible tools that offer in-depth analysis without the need for computational skills are lacking for biologists. Here, we present PatternJ, a novel toolset to analyze regular one-dimensional patterns precisely and automatically. This toolset, to be used with the popular imaging processing program ImageJ/Fiji, facilitates the extraction of key geometric features within and between pattern repeats in static images and time-lapse series. We validate PatternJ with simulated data and test it on images of sarcomeres from insect muscles and contracting cardiomyocytes, actin rings in neurons, and somites from zebrafish embryos obtained using confocal fluorescence microscopy, STORM, electron microscopy, and brightfield imaging. We show that the toolset delivers subpixel feature extraction reliably even with images of low signal-to-noise ratio. PatternJ's straightforward use and functionalities make it valuable for various scientific fields requiring quantitative one-dimensional pattern analysis, including the sarcomere biology of muscles or the patterning of mammalian axons, speeding up discoveries with the bonus of high reproducibility.

## INTRODUCTION

Regular patterns are a very common type of organization in animals. The division of the animal body plan into regular body segments is crucial for animal development from flies to humans (Hubaud and Pourquié, 2014; Nüsslein-Volhard and Wieschaus, 1980). Within each segment, bristles organize regularly at the surface of adult insects (Lawrence et al., 1979; Orenic et al., 1993), as feathers do on the skin of birds (Haupaix et al., 2018), or scales on the skin of reptiles (Chang et al., 2009). At the cellular scale, regularly repeated actin rings are found in mature axons (Leterrier et al., 2015; Xu et al., 2013), and actin organizes with myosin into periodic repeats in cell lines grown on rigid surfaces *in vitro* (Hu et al., 2017). Most impressively, in muscles, proteins organize in periodic sarcomeres that repeat themselves very regularly up to a hundred thousand times along a single myofibril (Lange et al., 2006; Luis and Schnorrer, 2021).

To understand how these regularly repeated patterns emerge over time, and how they are affected by mutations or environmental changes, it is important to precisely extract various important geometrical features, such as the size of a single pattern, its shape, or other geometrical arrangements at the level of a single pattern. Such quantitative extraction from images is often wanted by biologists. However, reaching this goal is often prone to error and bias when done manually, and it can be very time-consuming for biologists without extensive experience in computer programming.

Several automated tools have been developed in recent years to respond to such a need. The muscle field has been particularly active, with about 10 tools published over the last decade (Czirok et al., 2017; Lee et al., 2015; Morris et al., 2020; Neininger-Castro et al., 2023; Pasqualin et al., 2016; Sala et al., 2018; Spletter et al., 2018; Stein et al., 2022; Toepfer et al., 2019; Zhao et al., 2021). These tools are centered on the use in muscles and their sarcomeres: some focus on muscle contraction and give as output the contraction state of the tissue (Czirok et al., 2017; Lee et al., 2015; Sala et al., 2018), some can segment single sarcomeres from an image and extract their sarcomere lengths (Neininger-Castro et al., 2023; Toepfer et al., 2019) while others extract a global sarcomere length from an image with little to no input from the user (Morris et al., 2020; Pasqualin et al., 2016; Spletter et al., 2018; Stein et al., 2022). Often the input image is expected to display the periodic repetition of isolated single bands, usually obtained by staining the sarcomeric Z-disk, a thin protein-rich region bordering neighboring sarcomeres. The simplest tools usually require limited to no coding knowledge (Pasqualin et al., 2016; Sala et al., 2018; Spletter et al., 2018; Stein et al., 2022), while the most developed ones usually require advanced programming knowledge to be implemented by the user (Czirok et al., 2017; Lee et al., 2015; Morris et al., 2020; Neininger-Castro et al., 2023; Toepfer et al., 2019; Zhao et al., 2021).

Currently, only sarcApp provides an in-depth analysis of pattern features (Neininger-Castro et al., 2023). While the other ones can provide at best the spatial periods of individual patterns, sarcApp can extract the position of staining edges within patterns with, to our knowledge, pixel precision, and offers several other analyses including the direction of patterns, the number of patterns (or sarcomeres) or myofibrils in an image and more. However, an important limitation is that despite the efforts to make it user-friendly, it still requires programming skills that are not compatible with most users’ knowledge. As it is based on deep learning, it will require a computer that is adapted to the task, including a powerful GPU. Hence, the field would greatly benefit from a tool that provides an in-depth analysis of pattern features found in static images or time-lapse series, with subpixel precision, necessary to analyze fine pattern perturbation, and that works directly, neither requiring any programming knowledge, nor a dedicated powerful computer.

In this manuscript, we present PatternJ, a novel toolset to extract geometrical features from images of regular patterns. This macro toolset for ImageJ/Fiji (Schindelin et al., 2012; Schneider et al., 2012) does not require any coding knowledge and is aimed to be particularly user-friendly. It can extract with subpixel precision the size of individual periodic pattern repeats. Within a pattern, it can extract the position of multiple bands, blocks, and the shape characteristics of a typical phalloidin actin staining in muscle, a block with a middle band, a very common structure in the muscle field that is important to quantify. The extraction of these simple geometrical features will address most quantitative needs when analyzing repeated one-dimensional patterns found in nature. The tool provides the image of an averaged pattern, which is very useful for visualizations, especially for low signal-to-noise ratio (SNR) applications, as averaging will reduce noise and potentially reveal features that are not possible to observe otherwise. Finally, PatternJ can analyze time-lapse series, providing semi-automated ROI selection and feature tracking over time. We tested the tool for robustness against low SNR, aperiodicity, and intensity fluctuation, and evaluated how it behaves with different user selections. We then tested it on images of repeated patterns obtained by us from muscles and by colleagues from various tissues with different imaging methods.

## PatternJ workflow

**Fig. 1.**
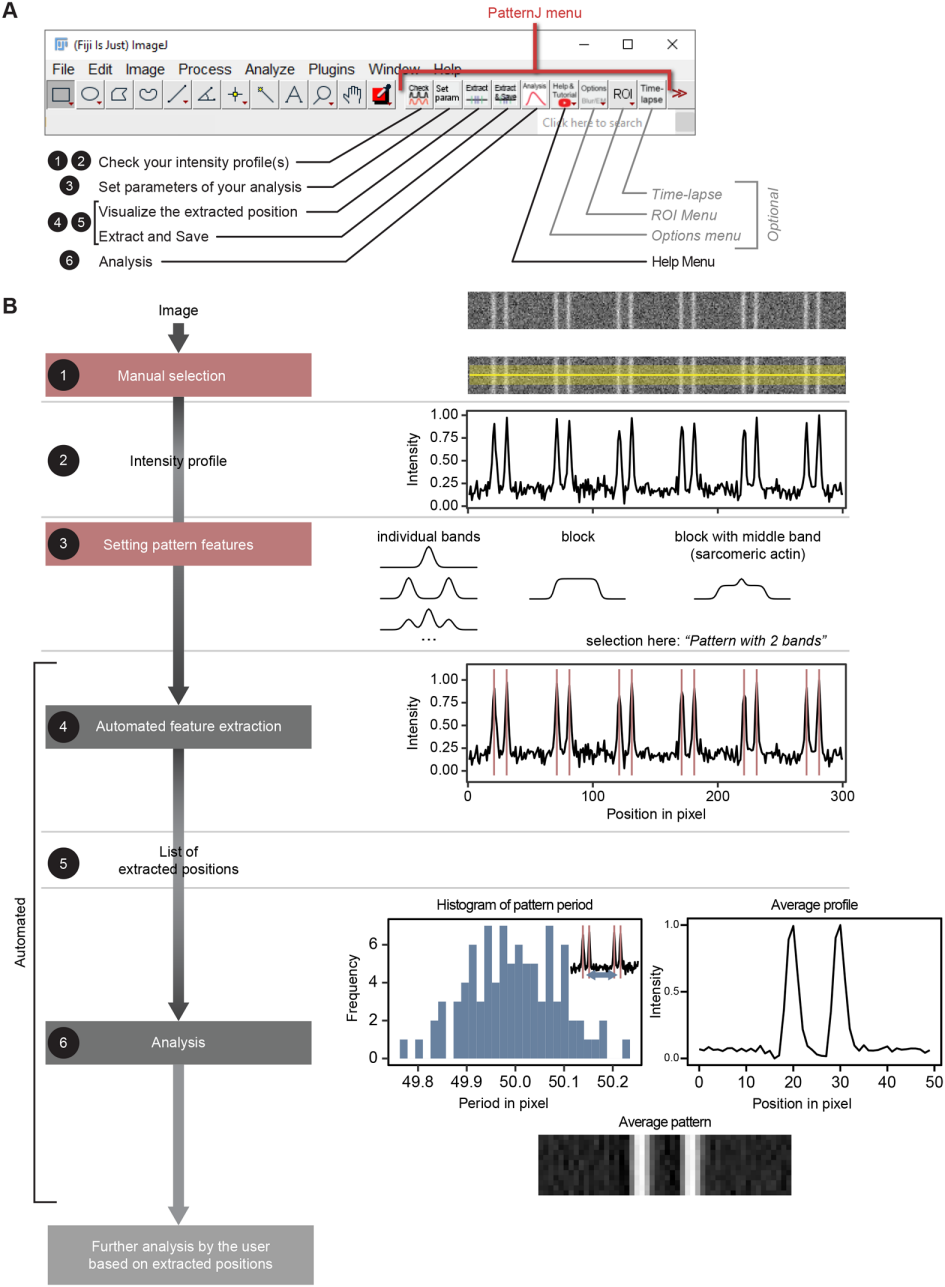
**PatternJ main features.** (A) Graphical user interface of PatternJ in Fiji and list of functions. The numbers 1 to 6 correspond to the steps presented in B. (B) Analysis steps followed by the user with the help of functions listed in A. A detailed description is found in the text.

The user will first install the toolset, which only requires copying the PatternJ files to the ImageJ macro toolset folder, as explained in the user manual and the video tutorial accessible on the PatternJ website at sites.google.com/view/patternj. After the installation, the toolset functions are accessible on the ImageJ/Fiji graphical user interface (Fig. 1A). The functions guide the user to analyze in a few steps the repeated patterns present in the selected image. The typical steps the user will follow are (Fig. 1):

1. **Manual selection**: draw a selection as a line or a curve on the image and check the corresponding intensity profile (“Check your intensity profile(s)” function). Multi-channel images will give a graph with one profile per color. Note that on straight selections, an error in the angle of the selection has only a small influence on the length measurements, as it scales with the inverse of the error angle cosine (see Fig. S1 for more details). In practice, a 5-degree error, which is obvious to the human eye, generates only a 0.38% error in the measurement.
2. Visually check the profile. The intensity of the profile is normalized, with the minimum value set to 0 and the maximum to 1, to allow for the comparison of multiple channels.
3. **Setting pattern features**: set the pattern characteristics through a popup window (“Set parameters of your analysis” function). The possible pattern characteristics are chosen between predefined patterns such as individual bands, blocks, or one block with a central band, as often observed in the muscle field for sarcomeric actin. The choice of pattern is asked only once. Note: strictly, a pattern is a recurring motif that repeats itself every pattern length along the profile. The pattern length can be easily defined, but the motif has an infinite number of ways to be defined: how to define the center of the motif for instance? To solve this challenge, the algorithm defines a pattern by having the features (bands, or blocks) centered on the pattern (Fig. S2). If the pattern consists of multiple bands (or blocks), the pattern configuration chosen will be the bands (or blocks) closest together, with the center position of the pattern as the average position of bands (or blocks); if the bands (or blocks) differ in intensity, the ones with the highest intensities will be placed toward the center. If the pattern is defined as a “block with middle band”, PatternJ defines the middle band as the center of the pattern.
4. **Automated feature extraction**: The algorithm automatically finds the pattern features based on the previous settings step and the user checks visually if they are found as expected (“Visualize the extracted position” function).
5. Once the features are correctly extracted, the user can save them (“Extract and Save” function). To analyze more regions of interest (ROI) on the image or image series, only steps 1, 4, and 5 would need to be repeated. To note, PatternJ saves the ROI selected. Previous ROIs can be retrieved with a dedicated function in the ROI menu of the toolset.
6. **Analysis**: After repeating these steps for multiple selections on one or multiple images, the user can then proceed to the analysis of the patterns (“Analysis” function). The function will compute the pattern period for each pattern individually, save it in a file, and concatenate the features extracted in separate files for each channel. Finally, it will compute from all patterns analyzed the average intensity profile of a pattern and generate an image of the average pattern.

The user can further analyze the positions of the pattern features extracted as they can be found in user-friendly generated text files. Additionally, the user can import previous ROIs, or ROIs generated by another algorithm and analyze them in a single step using a dedicated function found in the ROI menu without further input.

In case the image type or quality makes the analysis difficult, we added two optional steps accessible in the “Options” menu. The first one uses a Gaussian blur on the image in case the image presents a very low SNR; we found it to be useful, although it slightly lowers the image resolution. The second option inverts the gray values of the image. This is particularly useful when working with images from electron microscopy, which present dark values in electron-dense regions. In fluorescence imaging, this will also be useful when the feature of interest is dark in comparison to the rest of the selection.

Beyond static images, PatternJ also allows for the analysis of time-lapse series. To this end, the dedicated function “Time-lapse” guides the user through several simple steps. After checking that the settings of the pattern features are correctly set, the user is prompted to draw a few selections of the same object at different time points of the time-lapse (Fig. S3). The number of selections needed will depend on the image series. If the end positions of the object do not move, two selections are sufficient. Otherwise, the user needs to draw a couple of selections per dynamic change of the structure, which is typically about one selection per 10 images or fewer. PatternJ then automatically generates the selections on skipped images with bilinear interpolation (see Materials and Methods for more details). Once the selections are all generated, PatternJ analyzes the image series as it does for single images, with no additional input from the user. The user selections and the PatternJ-generated ones are saved in two files that can be easily imported later if needed. After PatternJ has extracted the position of features, these are tracked over time and displayed in a graph, together with the pattern size distribution over time and a stack of profiles with the position extracted for each timepoint and each channel (Fig. S3).

In case the user needs support to navigate the tool, we added a “Help” menu that provides a basic tutorial and directly links to the PatternJ website and to our video tutorials.

Automated feature extraction is the core of the tool. The algorithm takes multiple steps to achieve this (Fig. S4):

**Step 1**: the intensity profile is used to obtain a first estimate of the spatial period by identifying the first secondary peak in the autocorrelation of the intensity profile. The first secondary peak position is first located with a one-pixel resolution with the local maximum function of ImageJ macro. Then, a small region around this local maximum is used to precisely locate the peak position by fitting a Gaussian curve, from which PatternJ extracts the center position of the Gaussian function fitted.

**Step 2**: this estimate of the spatial period is then used to automatically segment the profile. Step 2a, the algorithm automatically selects a region that is defined as a single reference pattern: the width of the region is the spatial period defined in the previous step and its center is the point of highest intensity in the profile. Step 2b, once the reference pattern is defined, it is used to find the other patterns hidden in the profile. The algorithm computes the cross-correlation between the intensity profile of the selection and the intensity profile of the reference pattern. Step 2c, the cross-correlation profile displays multiple peaks, some of which correspond to the center position of patterns. To select the correct peaks, the algorithm uses the peak of the highest intensity (the reference peak) as a starting point and then searches for a new peak in a search window starting half a spatial period away from the reference peak, with a spread of a full spatial period. This choice aims at the robustness of the algorithm; however, it limits the variation of the pattern size from 50% to 150% of the average pattern size. Once new peaks are found, the algorithm propagates this search, looking for new peaks one spatial period away from the previous peak. Step 2d, the search results in the full segmentation of the patterns in the profile. Note that in Step 2, the localization of peaks is obtained with the local maximum function of ImageJ macro, limiting the precision to one pixel, which is not limiting at this stage.

**Step 3**: with the patterns segmented, the algorithm can then precisely extract features in each of the patterns individually, reaching subpixel precision by fitting with pattern-specific functions. If “individual band(s)” is selected, the algorithm searches for the number of bands indicated by the user, fits a Gaussian function separately on each band, and uses the center position parameter of the Gaussian function to deduce the precise center position of the band (Fig. S5). If “block(s)” is selected, a sigmoid function is used to find the edges of the block. By default, PatternJ gives the edge as the inflection point of the fit (in other words the position at which 50% of the maximum is reached, which is a parameter of the sigmoid function). If the user wants to use another threshold, the parameters of the fit are given as an output of PatternJ, and a simple calculation can then be done to deduce the position for any desired threshold (Fig. S5). If “block with middle band (sarcomeric actin)” is selected, the position of the middle band is found by fitting a Gaussian function and using the center of the function. To extract the positions of edges, we first separate the pattern into two left and right parts, avoiding 10 pixels at the center of the pattern. Then on each of the two sides, we estimate the 90% quantile value. From this value, we extract the position at which the profile reaches half of this value, going up for the left edge or going down for the right edge (Fig. S5). This choice aims at the robustness of the algorithm.

From the user perspective, the PatternJ workflow is expected to be achieved within 1 min. The automated part of the algorithm should extract features with subpixel accuracy in a multi-channel image within 1 s or less on a typical laptop. The main input expected from the user is a manual selection on the image, which is quick and straightforward.

## RESULTS

## Validation of PatternJ

The steps used in the algorithm are aimed at making the feature extraction robust in a large range of situations, including challenging images, in which the signal-noise-ratio (SNR) is low, or which contain not very regular patterns. To test the behavior of the algorithm, we generated simulated images, for which their features are known, and we varied the SNR and the position of the pattern to simulate a range as wide as possible of situations users may face.

**Fig. 2.**
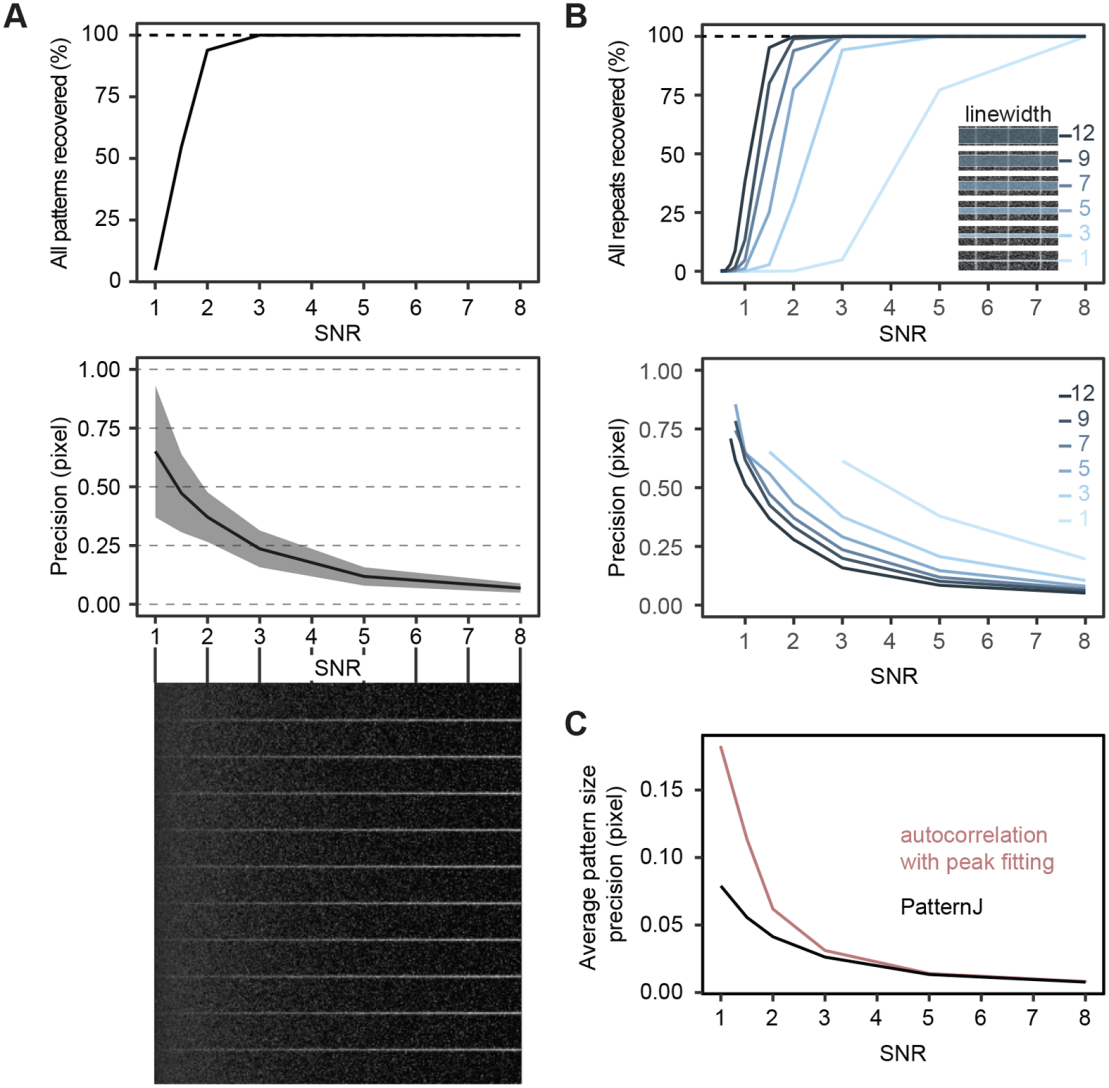
**PatternJ validation on patterns with varying signal-to-noise ratio (SNR).** (A) PatternJ performance when analyzing a single band pattern with varying (SNR). Top, fraction of selections in which all patterns were recovered. Middle, average precision in extracting the band position (line: average error in pixel, gray area: +/- standard deviation of the error in pixel). Bottom, examples of repeated patterns with varying SNR. (B) Effect of the selection linewidth on PatternJ performance on recovery and precision. (C) Comparison of the precision of PatternJ and autocorrelation in extracting the pattern period. See Materials and Methods for details on image generation and analysis.

We started by generating images with a single band pattern repeated 10 times, separated by 25 pixels (Fig. 2). All bands have the same intensity and Poisson-generated noise is added gradually to modify the SNR from 0.5 to 8. We generated 1000 images for each SNR value selected. To obtain a first estimate of the algorithm capabilities to extract the position of the bands, we used a linear selection encompassing the 10 patterns with a linewidth of 7 pixels. We evaluated the fraction of images from which all bands were correctly extracted (Fig. 2A). At SNR=1, it is very difficult to visually locate bands and the algorithm extracted all 10 bands in only 5% of images. However, already at SNR=1.5, which is still very challenging visually, all 10 bands were extracted in 55% of images. At SNR=2, all bands were recovered in 95% of images. For higher SNR, all bands were recovered in all images (Fig. 2A). Next, we estimated the precision at which the algorithm extracted the position of a single band (Fig. 2A). Even in very challenging SNR conditions, the bands were found with subpixel precision. At SNR=3, one can expect a precision of at least a quarter of a pixel, and a tenth of a pixel or better for higher SNRs. Overall, in these first examples PatternJ can extract robustly the positions of single bands even in challenging SNR conditions with a localization precision below a pixel.

Since the result of the extraction is likely to depend on the linewidth defined by the user, we changed the linewidth of the selection using the same images (Fig. 2B). The recovery and, to a lesser extent, the precision were indeed affected by the linewidth chosen. With the small linewidths, the recovery and precision are reduced; however, the algorithm can still be usable in many situations a user may face. At larger linewidths, the recovery was noticeably better, even at SNR=1. For a linewidth of 12 pixels and SNR=1, the 10 bands were fully recovered in about 40% of images. We also evaluated how the algorithm would behave with increasingly shorter selections. We found that the selection length has little influence on the performance of feature extraction (Fig. S6A). Altogether, we find that the algorithm robustly extracts our test pattern with high precision, even in challenging SNR conditions. Depending on the application, the user will benefit from selecting a larger linewidth, as the largest linewidths ensure the best results.

One-dimensional single-band patterns are common in images of biological samples. To analyze such patterns, it is common to manually extract the first secondary peak of the intensity profile autocorrelation or Fast Fourier Transform (FFT) to estimate the average spatial period. This analysis is usually limited to selecting the position of the peak's highest value, which constrains the precision to one pixel. We already demonstrated that our algorithm achieves subpixel precision at the level of a single pattern. However, we were curious as to how PatternJ would compare to the autocorrelation approach if we would estimate the spatial period by precisely extracting the secondary peak of the autocorrelation function using a local Gaussian function fit; note that this improved approach is not readily accessible in Image/Fiji, but it can be achieved by writing custom programs. We found that PatternJ precision in estimating the spatial period of a pattern surpasses the improved autocorrelation approach, in particular at low SNR (Fig. 2C; Fig. S6B). The autocorrelation gives more weight to brighter bands, which biases the estimation of the mean, whereas PatternJ gives the same weight to each band, limiting bias in the mean estimation, hence the higher precision in estimating the spatial period. Therefore, we would advise using PatternJ over approaches using autocorrelation when estimating the average pattern size in which the pattern consists of a single band.

**Fig. 3.**
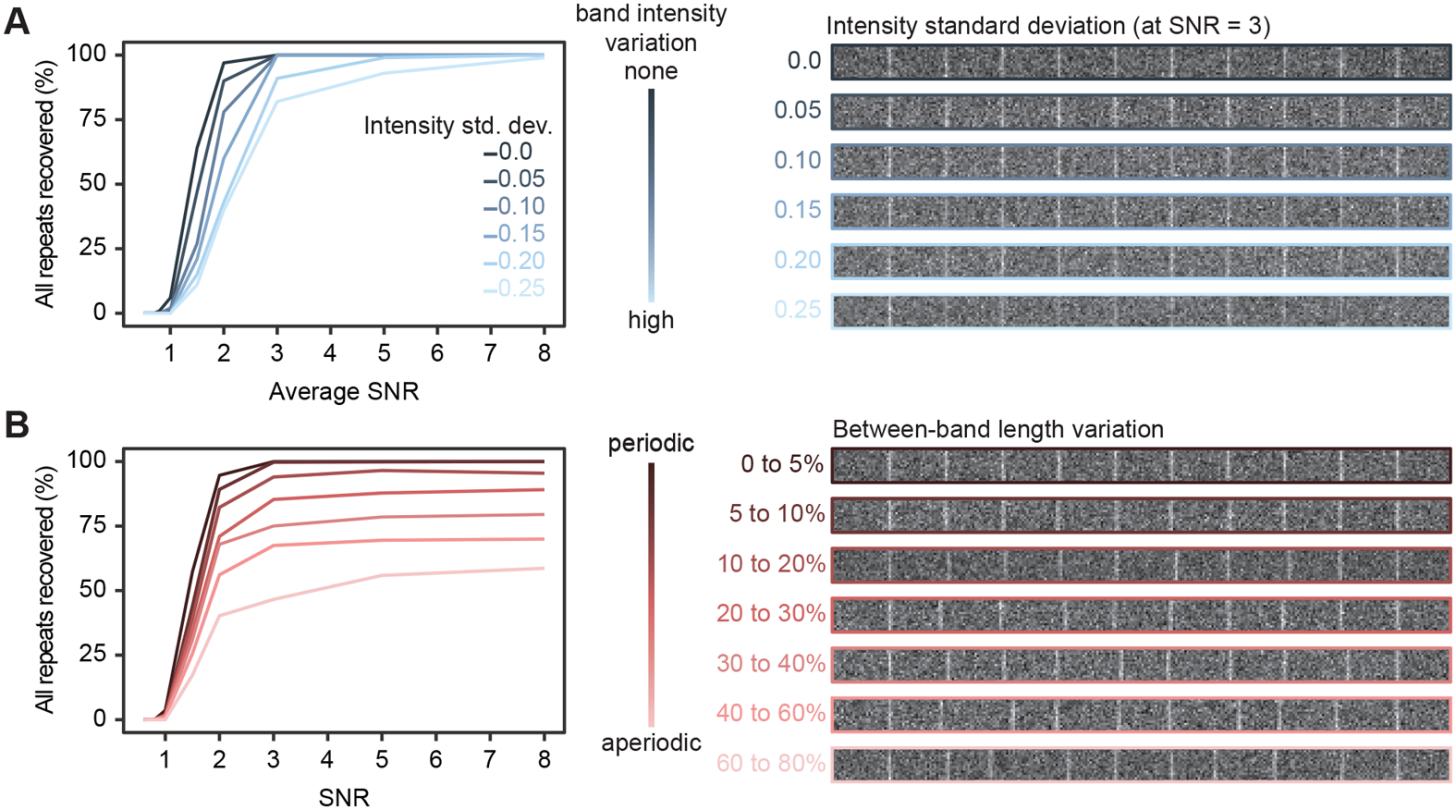
**PatternJ validation on patterns with varying intensity and periodicity.** (A) Left: effect of the band intensity variation on PatternJ performance on recovery. Right: image examples with varying band intensities. (B) Left: effect of the band length variation on PatternJ performance on recovery. Right: image examples with various degrees of between-band variation. See Materials and Methods for details on image generation and analysis.

Images of biological samples of course contain more complexity than the first examples we examined. To encompass more complexity, we kept a single-band pattern and additionally varied the intensity for all bands as well as their spatial period. By varying the band intensity, we found that the band recovery was more affected by the highest level of intensity variation (Fig. 3A). We suspect that bands of lowest intensities represent the bottleneck in this analysis: at a given average SNR, these bands have a lower SNR than the other bands, which makes them more challenging to extract. When varying the spatial period, the algorithm recovered patterns well up to 20% of variation in length, at which all 10 patterns were recovered in at least 90% of images, especially at high SNR (Fig. 3B). At higher variations of the spatial period, where the patterns are visually less periodic, the algorithm had more difficulty in extracting patterns. As this algorithm is built to be robust for repeated patterns, this result is not surprising. However, the algorithm can still be useful in these situations. Additionally, the user must check visually the output of the algorithm, which is a default feature of PatternJ. An algorithm using a peak finder function may be better suited when patterns become aperiodic, but it is likely to require more input from the user to be reliable.

## Comparison of PatternJ to existing tools

Several tools to analyze repeated patterns with different aims have been published in recent years (Table 1). SarcOptiM (Pasqualin et al., 2016) and MyofibrilJ (Spletter et al., 2018) use FFT to analyze an entire selection or the entire image and compute one value for the spatial period for the entire selection or image. SotaTool (Stein et al., 2022) uses a gray-level co-occurrence matrix to achieve the same result, with the addition of finding the sarcomere orientation automatically. Thus, these tools do not take into account potentially more complex patterns. SarcApp (Neininger-Castro et al., 2023), Sarctrack (Toepfer et al., 2019), Sarc-Graph (Zhao et al., 2021), and ZlineDetection (Morris et al., 2020) give access to the length of individual sarcomeres. SarcApp uses deep learning to extract the features of complex patterns, which requires some programming skills and a computer compatible with deep learning. Sarctrack, Sarc-Graph, and ZlineDetection can analyze movies of moving patterns, but they cannot extract complex features. Sarctrack and Sarc-Graph do not offer a graphical user interface (GUI), and although ZlineDetection, has a GUI, it requires a minimum of coding knowledge to run it on the user's computer. Except for SarcOptiM, all these tools do not require a manual selection from the user. Compared to these existing tools, PatternJ is very simple to use thanks to the GUI of ImageJ/Fiji and does not require programming skills. It can extract the position of complex features and the length of a single pattern. Additionally, it can combine the extraction results of multiple selections and display the distribution of spatial periods. Finally, it computes an image of the average pattern. The current limitation of PatternJ is the requirement of the user to draw manually a selection, although this step is a great part automated for the analysis of time-lapse series. In conclusion, we believe our tool provides a useful, user-friendly solution to extract simple and complex features from biologically relevant patterns.

**Table 1.**
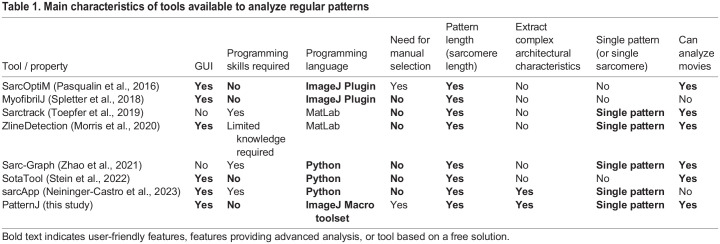
Main characteristics of tools available to analyze regular patterns

## Applications of PatternJ to biological samples

We validated our tool on simulated data, thus observing how it can analyze real data is important. To this end, we tested our tool with images acquired with various imaging methods in a wide range of tissues.

**Fig. 4.**
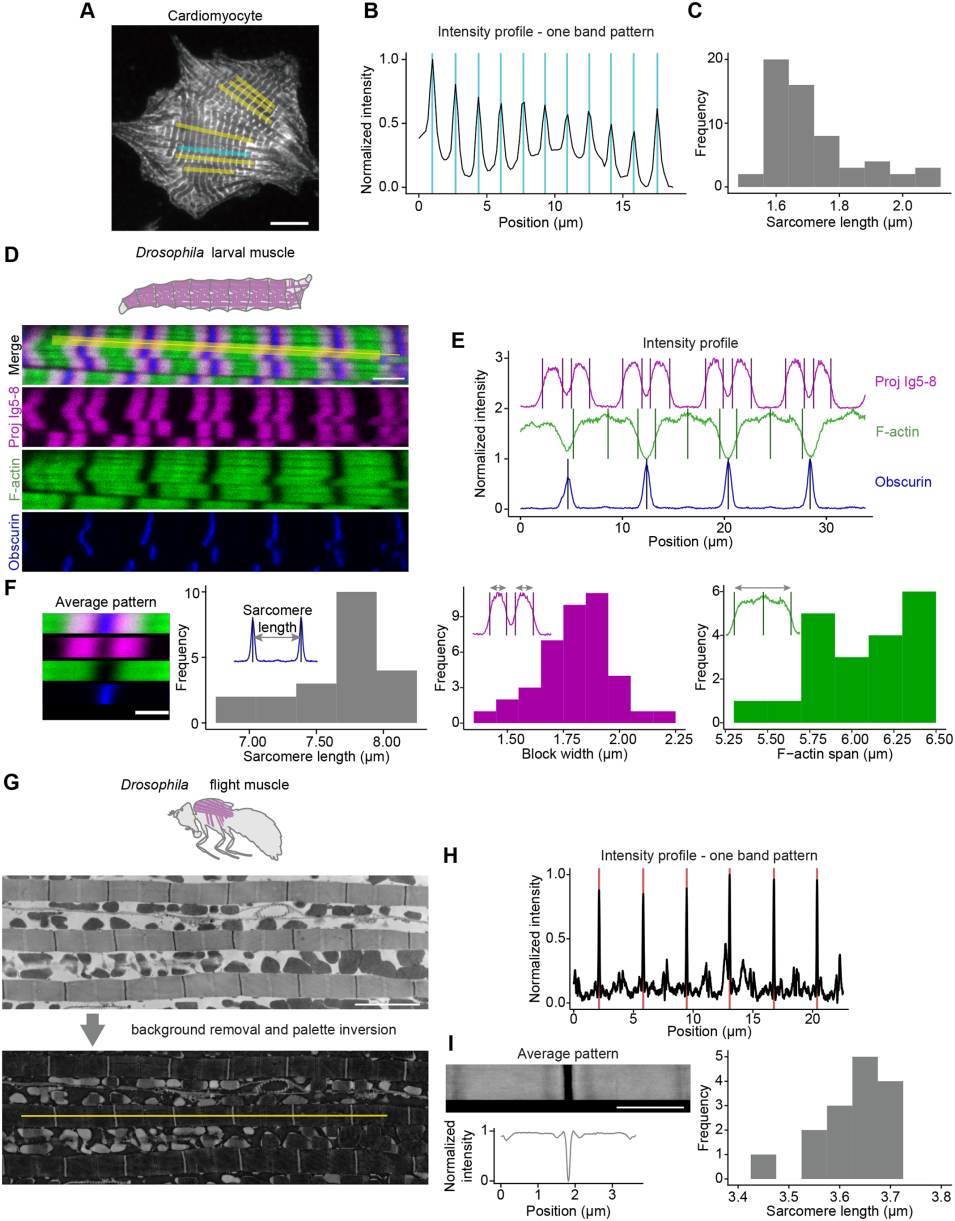
**Application of PatternJ to images of muscle samples.** (A) Confocal image of a cultured rat cardiomyocyte labeled for α-actinin; adapted from (Taniguchi et al., 2009). (B) Intensity profiles obtained at the cyan selection in A. Vertical bars correspond to the positions of bands extracted by PatternJ. (C) Distribution of sarcomere lengths from the yellow and cyan selections in A. (D) Confocal image of a *Drosophila* larval body wall muscle with labeled anti-Projectin nanobody [magenta, Proj-Ig5-8, Nano30 (Loreau et al., 2023)] and F-actin (phalloidin, green), as well as expressing GFP-tagged Obscurin (blue). (E) Intensity profiles obtained at the yellow selection in D. Vertical bars correspond to the positions of bands or blocks extracted by PatternJ. (F) Results of the analysis of six selections in the confocal image series of the larval muscle. Top left: image of the average pattern and average intensity profiles in each channel. Top right: distribution of the sarcomere length obtained from the position of Obscurin. Bottom left: distribution of the width of Projectin localization. Bottom right: distribution of thin filaments (F-actin) domain spanning. (G) Top: transmission electron microscopy image of adult *Drosophila* flight muscle (Loison et al., 2018). Bottom: same image background-corrected and with inverted pixel values. (H) Intensity profiles obtained at the yellow selection in G. The red vertical lines correspond to the positions extracted by PatternJ. (I) Left: image of the average pattern and average intensity profile (in both cases the intensity levels were inverted to display like a traditional electron microscopy image). Right: distribution of sarcomere lengths from the three myofibrils in G. Scale bars: (A) 10 µm, (D) and (G) 4 µm.

The main application of PatternJ will be to analyze images of muscle sarcomeres, from which extracting the position of proteins or protein domains is crucial to understanding how muscles are organized. We started by using our tool on confocal immunofluorescence images of cardiomyocytes in which sarcomeres were labeled for α-actinin (Taniguchi et al., 2009) (Fig. 4A). α-actinin is located at Z-disks displaying a single band (Fig. 4A,B). PatternJ can extract all positions of α-actinin on the selections chosen, from which it extracted the sarcomere lengths (Fig. 4B,C). Using the subpixel capabilities of PatternJ, the distribution of sarcomere length shows a variety of contraction states within the cell. This first biological example shows that PatternJ can extract robustly a biologically generated single band pattern with high precision.

We continued with using a multi-channel confocal image series of a *Drosophila* larval body wall muscle (Fig. 4D), in which Obscurin-GFP is expressed, and sarcomeres were labeled for Projectin and F-actin (Loreau et al., 2023). These proteins display a variety of features: Projectin staining consists of two blocks, F-actin is a block with a bright band in its middle, Obscurin is a single band on the M-band (Fig. 4D). PatternJ extracts the edge of each of the block-like shapes and the position of the center of each band (Fig. 4E). Moreover, the tool provides an image of the average pattern, an average intensity profile, and the distribution of sarcomere length (Fig. 4F). From the positions extracted, we could additionally obtain the width of Projectin localizations and the length of the actin filaments (F-actin) (Fig. 4F). This example shows that PatternJ can reliably extract features or patterns that are more complex than a single band. The analysis of a multi-channel image opens the possibility to observe how features in different channels vary at the pattern level.

Electron microscopy is another frequent way of imaging the organization of muscle. However, the images obtained are rarely quantified, because such images are challenging to analyze. We used an image of an adult *Drosophila* flight muscle (Loison et al., 2018) (Fig. 4G). These images display a very clear sarcomeric pattern, with the protein-rich Z-disk highlighted as a dark band. To extract the position of the Z-disk with PatternJ, one has to invert the image lookup table, so that it is treated as a bright band, similar to the previous examples using fluorescence microscopy. After the inversion and background correction, the positions of Z-disks are well extracted (Fig. 4H). PatternJ provides an average image of the pattern, an average intensity profile, and the distribution of sarcomere length (Fig. 4I). Here we used a dark feature of the image; however, a given image may display both lighter and darker features that will need to be extracted separately, one without inversion of the lookup table and one with inversion. We show with this example that PatternJ is not limited to the analysis of images obtained by fluorescence microscopy.

**Fig. 5.**
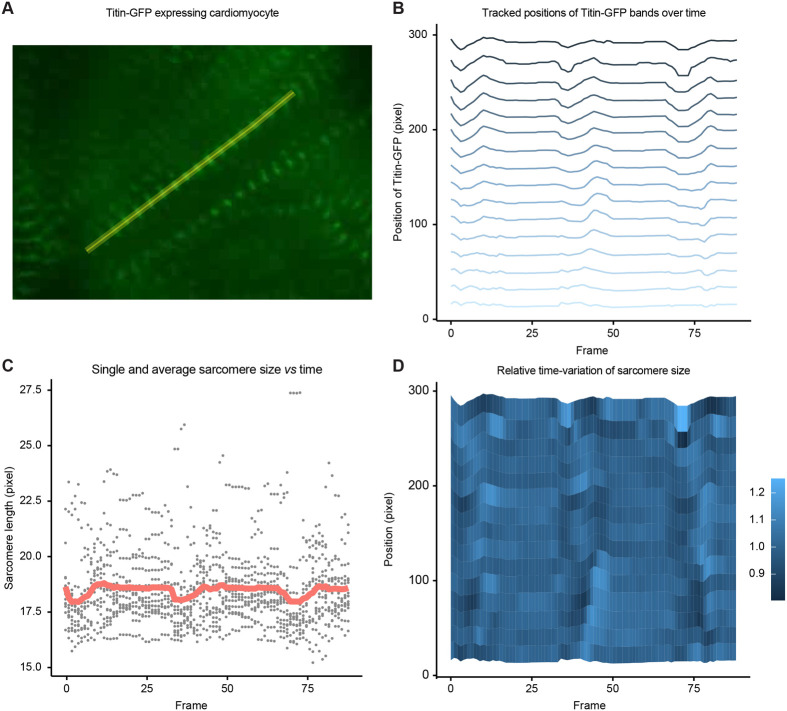
**Application of PatternJ to a time-lapse series of a contracting cardiomyocyte.** (A) Image of a cultured rat cardiomyocyte expressing Titin-GFP adapted from (Toepfer et al., 2019). One of the selections used by PatternJ is depicted in yellow. In this very dynamic movie, we have drawn manual selections in 14 frames for a total of 89 frames; PatternJ determined selections in frames without drawn selections. Note that the pixel size is not known to us, so we used pixels as scale units. (B) Tracked position of bands over time extracted by PatternJ. (C) Individual sarcomere lengths extracted at each time point (grey dots) and average sarcomere size over time (red path). (D) Quantification of the variation of sarcomere length over time. In this representation, the variations are computed independently for each sarcomere. The color scale depicts the variation of the sarcomere over time, which varies typically between 0.8 to 1.2, with 1 being the average sarcomere size over the 89 frames used here.

The analysis of the organization of proteins in static muscles is useful, however, PatternJ can also be used to track protein patterns over time. During muscle contraction sarcomeres shorten, with the myosin filaments sliding over actin filaments (Huxley and Hanson, 1954; Huxley and Niedergerke, 1954). Such contraction can for example be observed in cultured differentiated cardiomyocytes. Here we used a movie of a contracting cardiomyocyte from (Toepfer et al., 2019) and analyzed the location of the N-terminal end of Titin, located at the Z-disk and displaying single bands on microscopy images (Fig. 5). PatternJ extracts the position of Titin N-term over time, allows for the tracking of the several bands (Fig. 5B), and provides the average sarcomere size over time (Fig. 5C). This analysis displays the obvious cyclic contractions of sarcomeres. Using the outputs of PatternJ, one can visualize temporal variations at the level of single sarcomeres, exhibiting the rich dynamics of cardiomyocyte contraction, including waves of contraction along the myofibril length (Fig. 5D). This analysis is not limited to single band patterns and by construction, it can be generalized to any of the pattern types PatternJ can analyze.

**Fig. 6.**
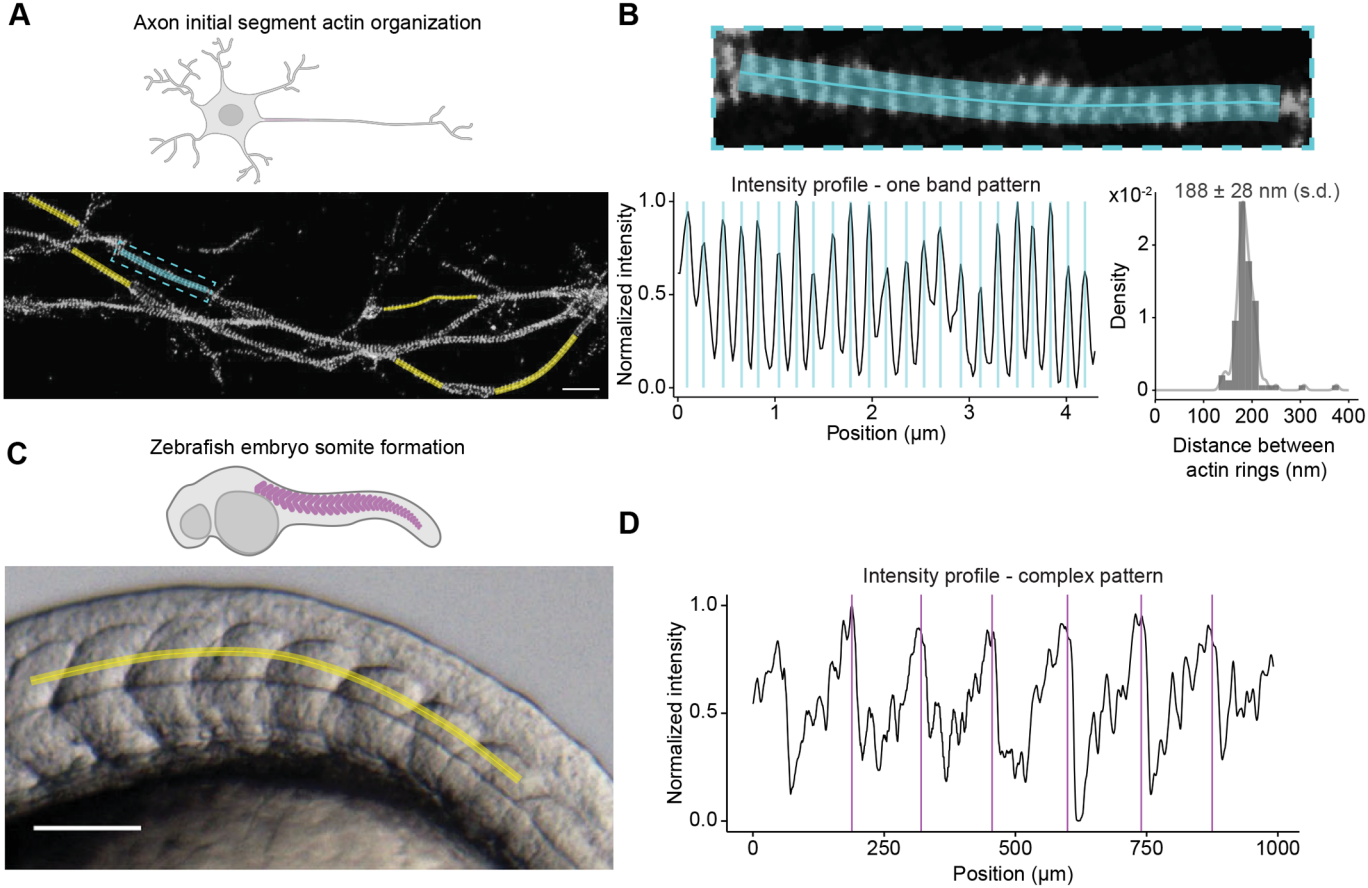
**Application of PatternJ to images of biological samples.** (A) STORM image of neurons, localizing βII spectrin adapted from (Zhong et al., 2014). (B) Top, zoom in on the selection highlighted in cyan in A. Bottom left, intensity profile obtained at the cyan selection in A. Vertical bars correspond to the positions of bands extracted by PatternJ. Bottom right, distribution of sarcomere lengths obtained from the cyan and yellow selections in A. (C) Brightfield image of a zebrafish embryo adapted from (Richter et al., 2017). (D) Intensity profile obtained at the yellow selection in C. Vertical bars correspond to the positions of patterns extracted by PatternJ. Scale bars: (A) 2 µm, (C) 200 µm.

Regularly spaced actin structures are not restricted to muscles. Actin rings were also observed in the axon initial segment (Leterrier et al., 2015; Xu et al., 2013). We tested PatternJ on super-resolved images of these actin rings obtained with the super-resolution method STORM (Fig. 6A) (Zhong et al., 2014). PatternJ extracts efficiently the position of individual actin rings. We find the typical 190 nm distance between actin rings that has been reported (Leterrier et al., 2015; Xu et al., 2013). Interestingly, we can also observe a significant variation around this value of about 28 nm (using the standard deviation, Fig. 6B). This variation of distance between rings can be noticed visually, potentially stemming from different protein organizations. This feature is rarely reported, because of a lack of tools to extract such data. Hence, we think our tool will be useful when analyzing such an organization. Having access to the variability of distances between rings will be particularly useful when studying perturbations of the organization. The transformation of a table of protein localization obtained from STORM or other pointillist methods into pixelated images makes the use of PatternJ straightforward for this type of super-resolution imaging technique.

Patterns can take very complex shapes, which are difficult to describe with a simple mathematical function, limiting the potential extraction of features. Such patterns are, however, important to analyze. A brightfield image of a zebrafish embryo is one example of such a situation: when extracting the intensity profile along formed somites, we obtained a very complex pattern, which is in part due to the presence of numerous granules (Fig. 6C). Our precise fitting algorithms for bands or blocks did not work in this condition. It is nevertheless possible to extract robustly the position of patterns (Fig. 6C), by using the second step of our algorithm, which identifies local maxima in the cross-correlation between the intensity profile and a single reference pattern profile. For this kind of situation, the extraction is not as precise as in the other examples, likely in the pixel range, but it is fully automated and it will be useful in situations in which many other algorithms would fail.

## DISCUSSION

PatternJ is a simple-to-use toolset for ImageJ/Fiji that can extract complex features in images of repeated patterns. It can even extract features from challenging images containing high noise, for which it outperforms the precision of FFT or autocorrelation-only approaches to determine the average spatial period of the pattern (Fig. 2). The tool performs well in situations in which the intensity of the pattern varies, or the variation of the spatial period does not exceed 20% (Fig. 3). The tool can extract geometric features of the sarcomeric pattern from muscle samples and other tissues displaying repeated patterns both using static images (Figs 4 and 6) or time-lapse series (Fig. S3 and Fig. 5). In time-lapse series, the tracking of patterns allows for the analysis of the dynamic changes of the structure studied (Fig. S3 and Fig. 5). Even if it is designed primarily for fluorescence microscopy images, PatternJ can also be used on images taken with electron microscopes or brightfield microscopes (Figs 4G and 6C).

Several tools to analyze images with repeated patterns already exist: what makes PatternJ particularly useful for the biological community? The main advantage of PatternJ over other tools is that it does not require any programming knowledge or any specific computer and yet, it can extract complex pattern features. In contrast to other tools that offer the automated selection of regions of interest, PatternJ does require the user to select the region of interest manually. However, regions that are selected automatically in other tools are likely to eventually require manual curation. Except for the manual selection, the other steps in PatternJ are automated. Compared to FFT or autocorrelation-only algorithms, above an SNR of 3, PatternJ is not advantageous if one is only interested in the average length of the spatial period. However, PatternJ provides the complete distribution of spatial period length, containing additional information to understand the variability in the pattern organization or even multiple modes in the length distribution that would otherwise be lost using only the average period length. Finally, to our knowledge, PatternJ is the only tool available that can extract complex pattern features from time-lapse series data.

What are the limitations of PatternJ? A first limitation appears in low SNR conditions when a pattern consists of multiple bands. Currently, PatternJ searches in a given pattern for the number of bands indicated by the user, by looking for local maxima in the intensity profile. However, in low SNR conditions, the noise generates local maxima that may be wrongly identified as bands. A simple solution to this limitation is to use Gaussian blurring, which is a built-in PatternJ function, or deconvolution for more advanced users: noise is then significantly reduced and multiple-band patterns are then usually well extracted. When possible, using a thicker linewidth in the selection can also eliminate such limitations as it reduces the effect of noise (Fig. 2B). We showed that electron microscopy images can be used, but since relevant information is usually in electron-dense regions, where pixel intensity values are low, it will be beneficial for the user to invert the pixel values to make them analogous to fluorescence images. After this simple step, which is included in PatternJ functions, pattern features will be extracted with high precision.

Thanks to its ease of use and its capabilities, PatternJ is aimed at being used by a wide range of biology users, in fields that study the formation of regular biological patterns. Its precision and automation make analysis fast, robust, and reproducible, which we hope will help speed up discoveries.

## MATERIALS AND METHODS

### *Drosophila* preparation for fluorescence microscopy

Larval muscles were stained with fluorescent nanobodies (Loreau et al., 2023) and rhodamine-phalloidin (1:1000 molecular probes) for 2 h at room temperature or overnight at 4°C as previously described in detail (Loreau et al., 2023). They were mounted in SlowFadeTM Gold Antifade (Thermo Fisher Scientific) and imaged with a Zeiss LSM880 confocal microscope using a 63× objective.

### *Drosophila* preparation for electron microscopy

The detailed protocol for how the electron microscopy of flight muscles was obtained was reported in (Loison et al., 2018). After staining and epon embedding, sections of 90 nm were cut on a Leica UC7 microtome and additionally stained with 2% uranyl acetate (Thermo Fisher Scientific, 6159-44-0) for 30 min and 0.4% lead citrate (Thermo Fisher Scientific, NC1588038) for 3 min to enhance the contrast. Images were acquired with a Zeiss EM 900 (80 kV) using a side-mounted camera from Osis (Loison et al., 2018).

### Simulations

Simulated images used for the validation of PatternJ were generated in Python. In brief, single bands were added to oversampled images, that were then blurred with a Gaussian function to simulate diffraction. After resampling to the pixel size of a typical microscope using a 100× objective (42 nm), we added Poisson noise to simulate electronic noise.

Images of varying band intensity were obtained in the following way. A given band has one value for its intensity, which is homogeneous in a band. When varying the intensity, bands take values for their intensity in a normal distribution, of mean value 1 and of standard deviation that depends on the simulation, ranging from 0 (all bands have the intensity 1) to 0.25 (band intensity typically takes value in the 0.5–1.5 range). To vary band positions, each band could shift its position by selecting randomly a shift amplitude in a normal distribution of mean 0 and of increasingly large standard deviation. After the generation of positions, images were classified based on the difference in percent of the distances between the two bands closest and the two bands furthest. The code used to generate these images is accessible at github.com/PierreMangeol/PatternJ.

A band was considered detected if the algorithm could find a band within 2.36 pixels from its actual position (corresponding to 100 nm at a 42 nm pixel size). Once bands were considered detected, we computed the average pattern size and the standard deviation of the pattern size. The precision of localization reported in Fig. 2 was obtained by averaging the standard deviation of the extracted pattern size for selections in which all bands were found. The standard deviation reported is the standard deviation of the standard deviation of the extracted pattern size for the same selections.

### Intensity displayed in PatternJ

All intensities displayed in the manuscript, as well as in the tool itself, are normalized in the following way: the minimum value in a profile is set to 0 and the maximum value to 1.

### Background correction for electron microscopy images

We found that background correction helps the algorithm reproducibility when used on electron microscopy images. PatternJ offers an optional step to achieve this. In this step the background is subtracted using the background subtraction tool of ImageJ/Fiji with a rolling ball radius of 50 pixels, with the additional parameters “light background” and “disable smoothing”; after background correction, the look-up table is inverted.

### Interpolation of selections for time-lapse

In time-lapse, PatternJ generates selections that are not drawn by the user. If a user draws a selection in frames *i* and *j*, PatternJ generates selections in all frames between the frames *i* and *j*. To this end, user-drawn selections are first interpolated with 100 points each. Let (Xi_1_, …, Xi_100_), (Yi_1_, …, Yi_100_) be the X and Y coordinates along the selection *i* and similarly (Xj_1_, …, Xj_100_), (Yj_1_, …, Yj_100_) the X and Y coordinates along the selection *j*. The coordinates of the selection in frame *i*+*k* is given by:

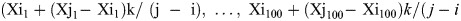


and

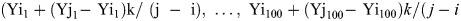


### Tracking of features in time-lapse

Once features are extracted, PatternJ links patterns from one image to another over time. A given pattern, regardless of how complex it is, is given one reference position. This position is computed as the average position of bands in a pattern, the average position of edges, or the position of the band if the pattern is a block with a middle band. PatternJ then tracks these reference positions over time. The principle behind the tracking algorithm is simple. From a given pattern found in the first frame, the algorithm searches for the closest reference with a maximum distance of half a pattern size in the next frame. If such a reference point is not found, the algorithm searches for it frame after frame, up to 20 frames. Because the pattern size is time-dependent, all sizes are rescaled with the length of the selection at a given time point. This prevents the range search from being too small when patterns are large or too large when the patterns are small.

### Availability

PatternJ can be downloaded from the PatternJ website at sites. google.com/view/patternj. The website also contains a manual and a video tutorial to install and use the tool. The coder community will also find the content at github.com/PierreMangeol/PatternJ.

## Supplementary Material

10.1242/biolopen.060548_sup1 Supplementary information
