# Supplementary material for: PatternJ: an ImageJ toolset for the automated and quantitative analysis of regular spatial patterns found in sarcomeres, axons, somites, and more
Source: Biol Open. 2024 Jun 18;13(6):bio060548. doi: 10.1242/bio.060548 (PMC11212633; doi:10.1242/bio.060548)
Supplement: Supplementary information [file biolopen-13-060548-s1.pdf]

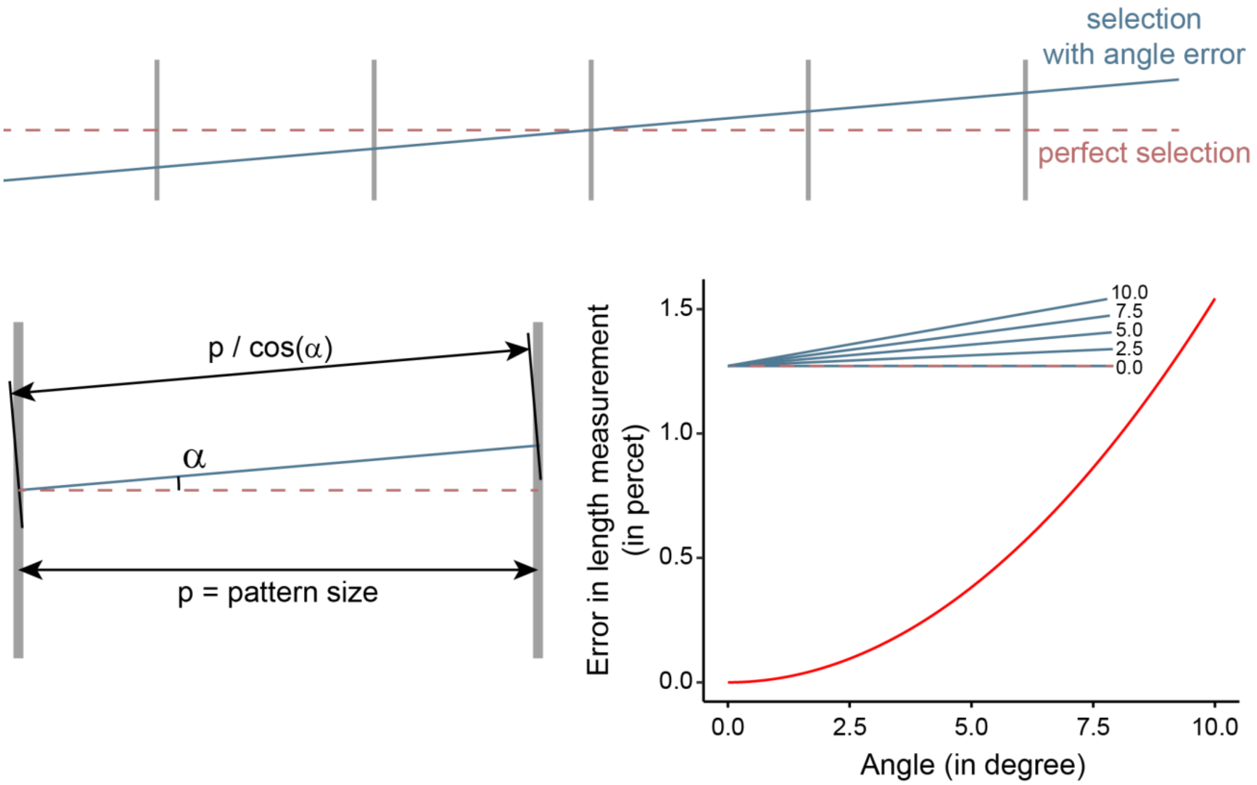

Fig. S1. Effect of the selection angle error on the length measurements. With *p* the pattern size, an error of angle *α* in the selection will generate a pattern size estimate of *p* / cos(*α*). The graph represents this function, with on the top left inset examples of such deviations in degrees. For an obvious error of 5 degrees, the pattern size error is below 0.5%.

Biology Open • Supplementary information

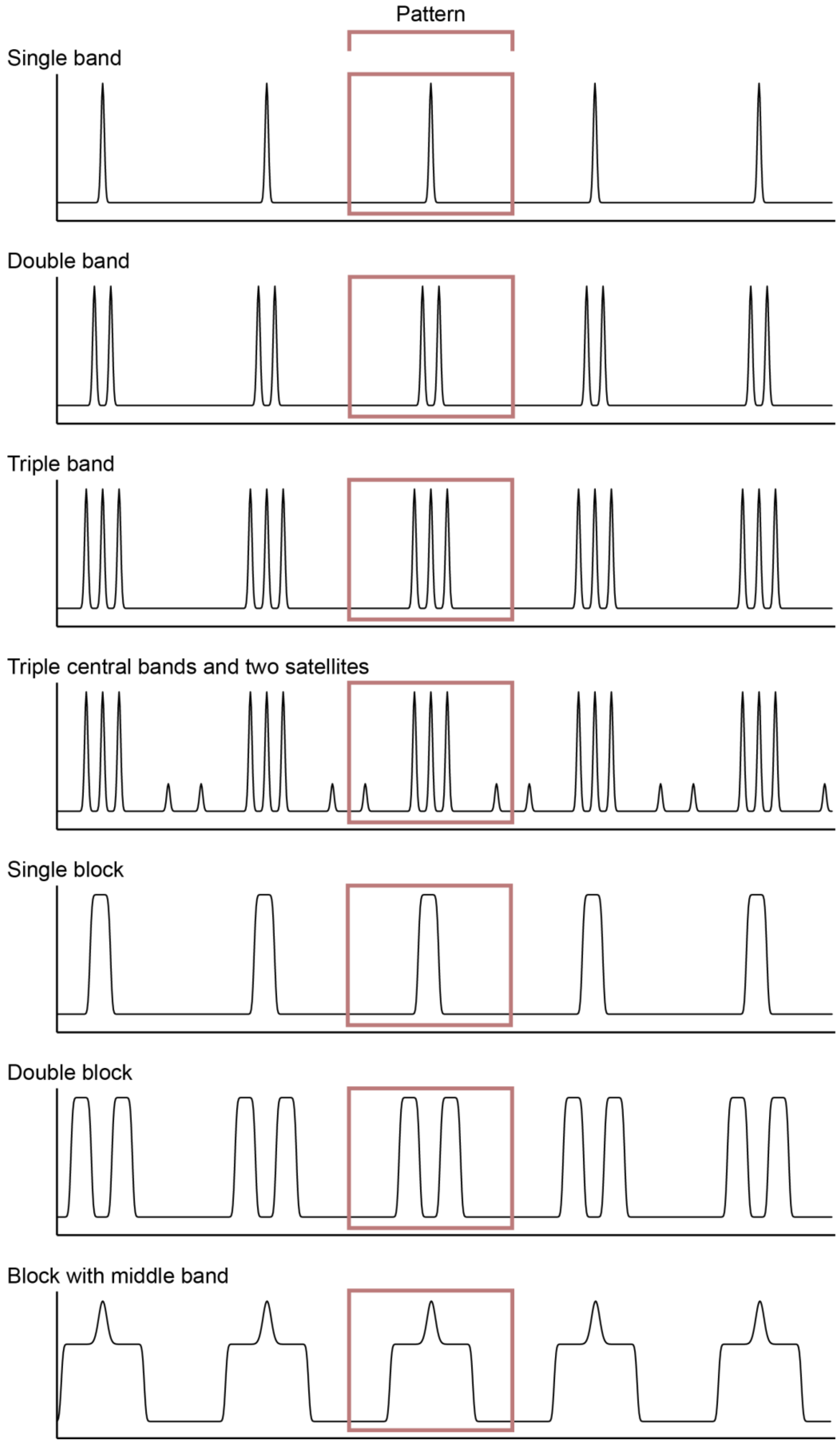

Fig. S2. Definition of a pattern on a few typical examples, with multiple bands, multiple blocks, or a block with a middle band.

Biology Open • Supplementary information

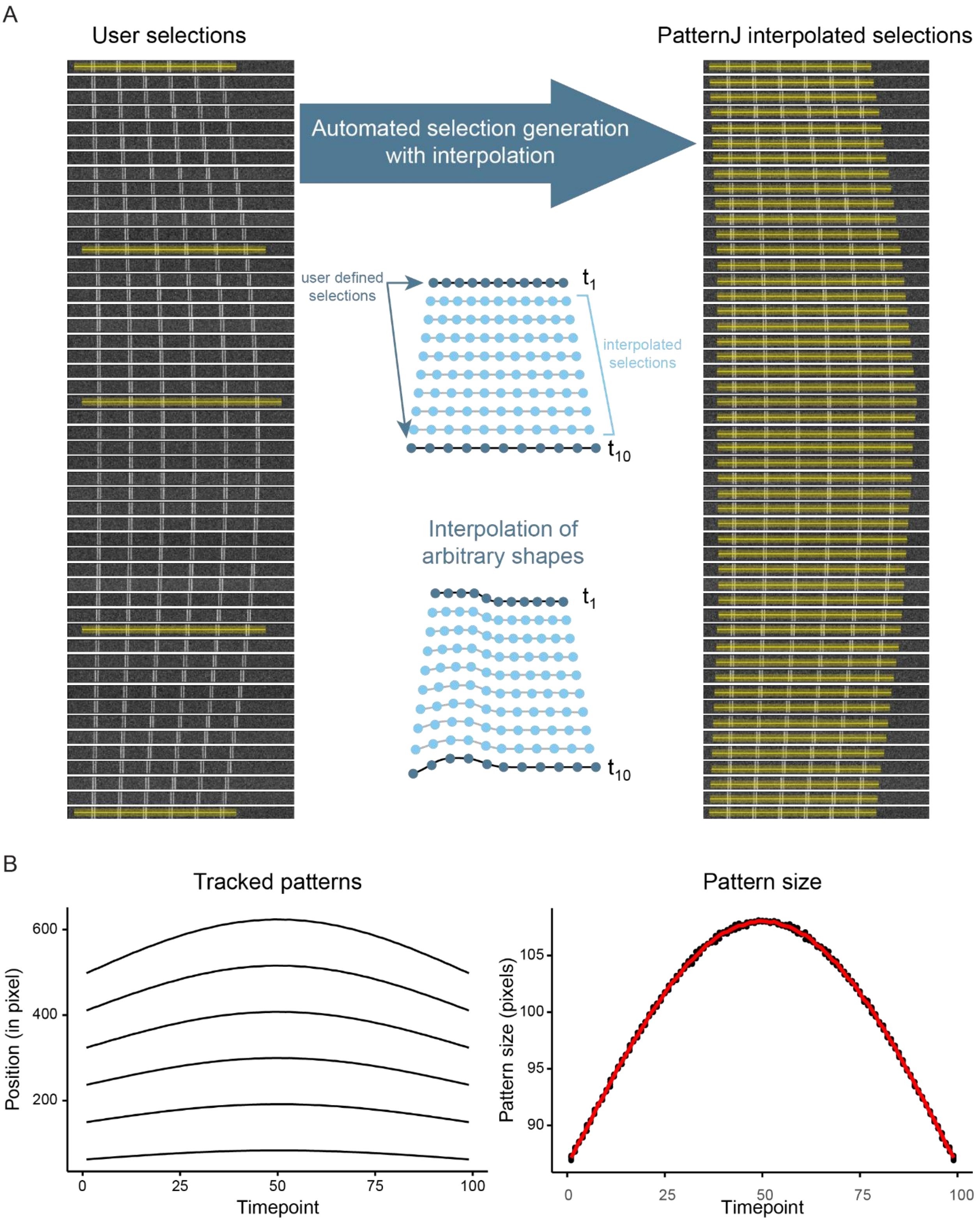

Fig. S3. Time-lapse analysis. (A) The user draws a few selections on the same object at different time points (left image sequence, yellow selections). PatternJ then interpolates these selections with 100 points, from which it defines the selections in frames in which no selection was drawn (right image, yellow selections). These selections are obtained by linear interpolation

Biology Open • Supplementary information

between the two user-drawn selections (see details in the method section). We provide an example of interpolation on linear segments (center top), however, this approach can be also used on any line shape (center bottom). (B) Outputs of the analysis: tracked pattern positions over time (or slice) and individual and averaged pattern size over time (or slice). The position of features in each pattern is also extracted for each time point. These are not represented here for simplicity.

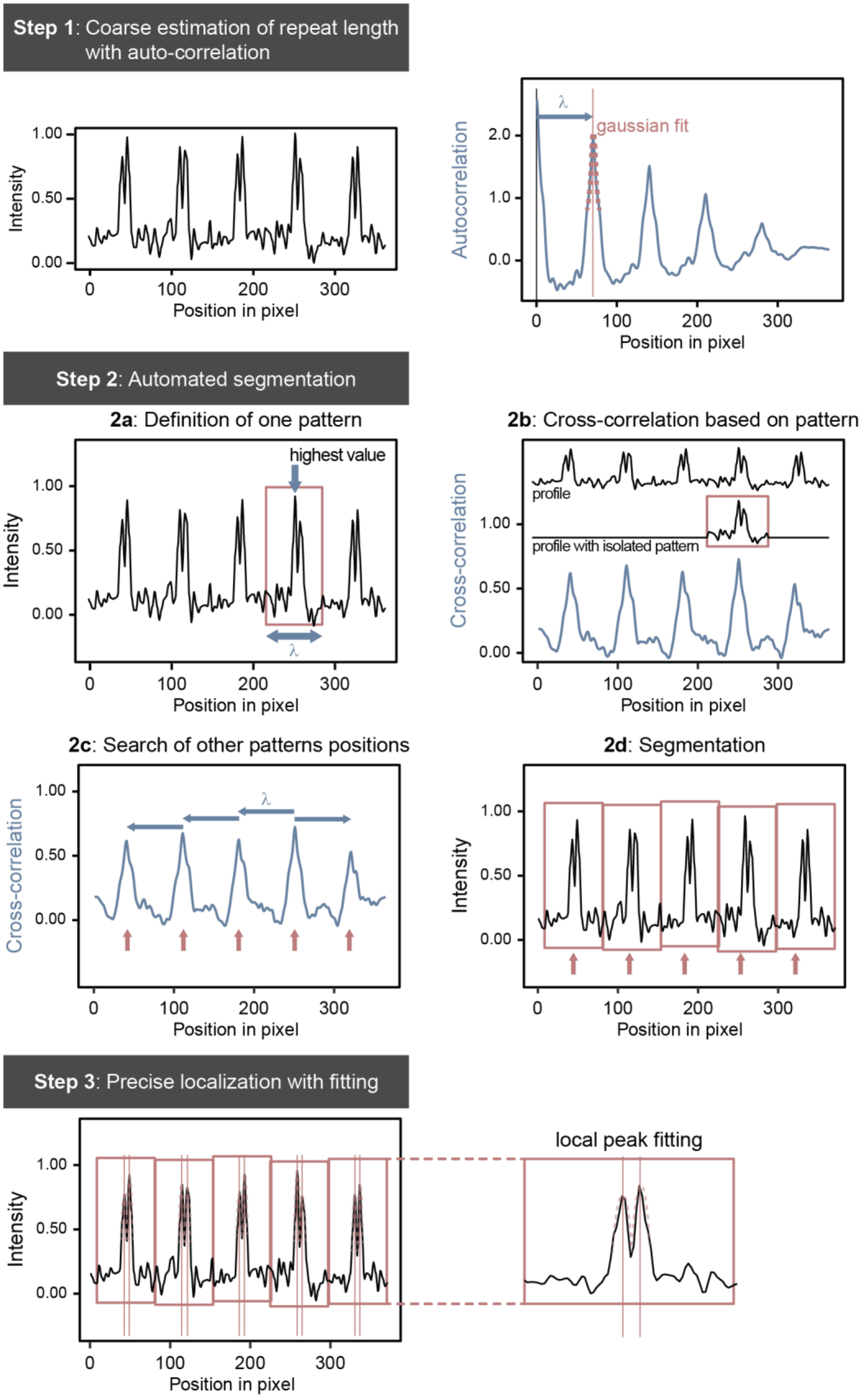

Fig. S4. Main steps followed by PatternJ to automatically extract pattern features precisely. A complete description is found in the main text.

Biology Open • Supplementary information

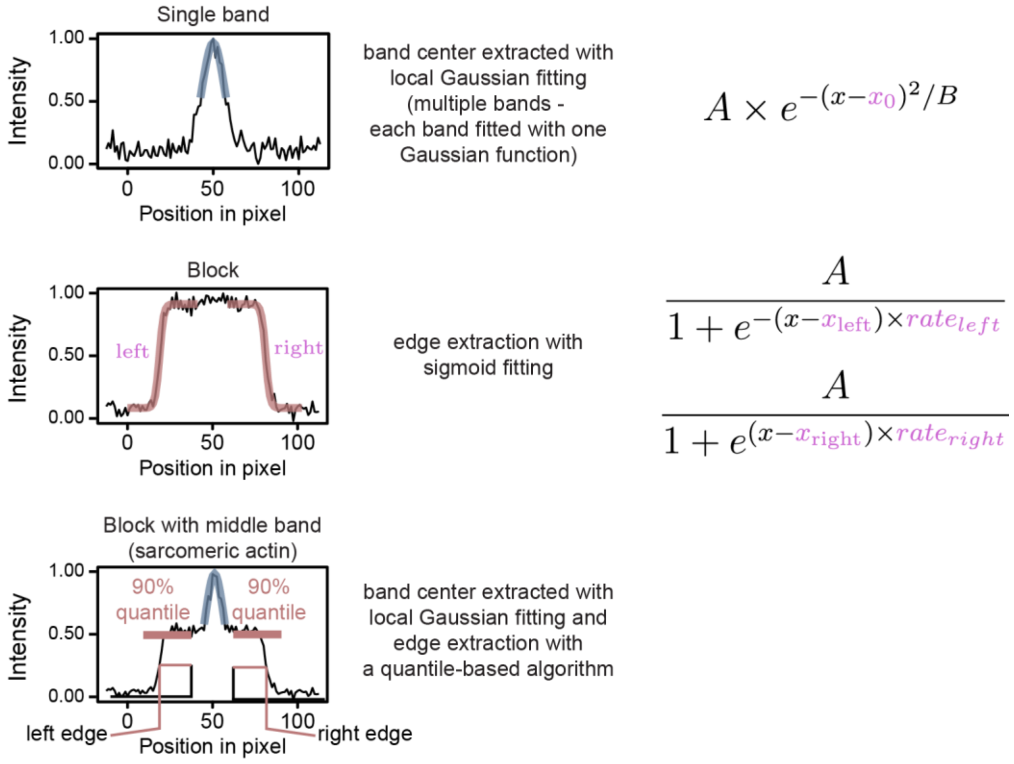

Fig. S5. Fitting procedures used to extract pattern features. Features identified as “bands” are fitted with a Gaussian function. “Blocks” are fitted with two sigmoid functions. The fit parameters in magenta are given as an output of PatternJ. For sigmoid functions, the fit parameter for the position (x_{right} and x_{left}) gives the position at the inflection point, which is the position at 50% of the full amplitude. The user can deduce the position at any fraction α of the maximum amplitude A with the formula $x_\alpha = x_{left} - \ln(1\text{-}1/\alpha)/rate_{left}$ for the left edge and $x_\alpha = x_{right} + \ln(1\text{-}1/\alpha)/rate_{right}$ on the right edge, with α between 0 and 1. The “Block with middle band (sarcomeric actin)” pattern is fitted by one Gaussian function on its center band and by an algorithm based on quantiles for edges: the 90% quantile value for intensity is estimated on the left and right of the pattern individually. From this value, the position at which the profile reaches half of this value is extracted and defines the edge position, increasing for the left edge or decreasing for the right edge.

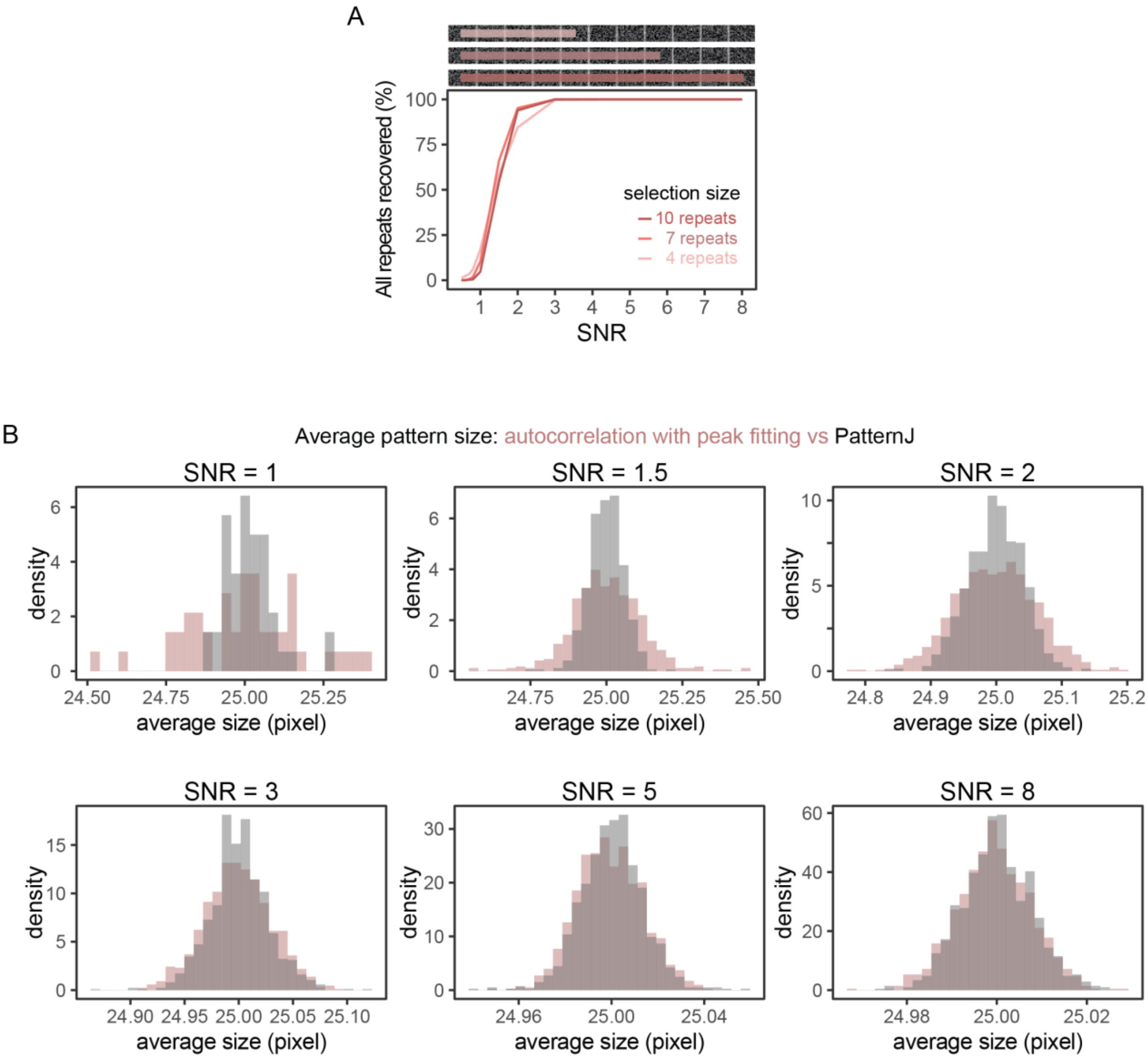

Fig. S6. (A) Effect of the selection length on the performance of PatternJ to extract all patterns from a selection. (B) Comparison of the precision of PatternJ and autocorrelation using peak fitting, for a range of SNR typically observed on images of biological samples.

Dataset 1.

Available for download at
https://journals.biologists.com/bio/article-lookup/doi/10.1242/bio.060548#supplementary-data

Dataset 2.

Available for download at
https://journals.biologists.com/bio/article-lookup/doi/10.1242/bio.060548#supplementary-data
